# Gender inequality in vision loss and eye diseases: Evidence from the Sultanate of Oman

**DOI:** 10.4103/0301-4738.57153

**Published:** 2009

**Authors:** Rajiv Khandekar, A J Mohammed

**Affiliations:** Eye & Ear Health Care, Department of Non-Communicable Disease Control, Director General of Health Affairs, Oman; 1 Advisor, Health Affairs, Ministry of Health, Oman

**Keywords:** Blindness, eye diseases, gender, Oman

## Abstract

**Purpose:**

The data from surveys of vision loss and monitoring of services were used to assess changes in gender inequality in Oman.

**Study Design:**

Retrospective review of data collection instruments.

**Materials and Method:**

The data sets of 12 years between 1996 and 2007 were abstracted to assess the gender equality for vision loss, eye disease prevalence, and service use. They included two surveys (1996 and 2005), Health Information from eye units (1998 and 2007), and eye screening in schools.

**Results:**

In 1996, the prevalence of bilateral blindness in ≥ 40 years of age was higher in females [Odd's Ratio (OR) = 0.36 (95% Confidence Interval (CI) 0.24 – 0.53)]. Gender differences in the prevalence of cataract [OR = 0.82 (95% CI 0.63 – 1.03)] were not significant while trachomatous trichaisis (TT) was less in males [OR = 0.33 (95% CI 0.22-0.48)]. In 2005, gender differences in the prevalence of bilateral blindness [OR = 0.97 (95% CI 0.71 – 1.34)] and TT [OR = 0.66 (95% CI 0.42- 1.04)] were not statistically significant. But males were associated with higher prevalence of cataract [OR = 1.26 (95% CI 1.00 – 1.59)]. Surgery rates for cataract, glaucoma and TT were not different by gender. More male compared to female patients with diabetic retinopathy were treated. Myopia was significantly higher in girls. Compliance of spectacle wear was higher in girls.

**Conclusions:**

Gender inequality for eye care seems to have reduced in the last 10 years in Oman. However, apart from TT and glaucoma patients the difference in service utilization by gender was not statistically significant.

The global inequality in blindness prevalence and the uptake of cataract surgery has been well-recognized.[1] While there is evidence of a male predominance of glaucoma, reports suggest that more men access glaucoma treatment than women.[2] Furthermore, the sequel of trachoma affect women more commonly than men while the utilization of surgical services for trichiasis varied by gender.[3,4] Women have a twofold excess burden of cataract, glaucoma, trachoma, age-related diseases.[5,6] Two population-based studies in Malawi (1983 and 1999) showed a reduction in blindness rates but no change in gender inequality.[7] To date, there has been no assessment of the impact of VISION 2020 in terms of gender equality which is one of the millennium development goals.[8]

Oman has a population of 2.5 million; 1.88 million of them are indigenous and the male to female ratio is 51:49. It has nearly 160 primary health centers with an integrated eye care service and 30 secondary eye care units. Services including surgeries and medicines are offered at an expense of less than US $ five per year. Secondary services are managed by 105 qualified ophthalmologists and 40 optometrists. VISION 2020-Oman was launched in March 1999.[9]

The health information and management system (HIMS) has a computerized network in all regions. The nurse and ophthalmologists provide information on morbidity of eye diseases as per ICD 10 codes and about their surgical managements. The annual data is monitored to ensure its quality. The Ministry of Health (MOH) publishes annual health reports that include demography, status of resources and these data.[10]

We conducted a review of the data of two national eye surveys and data on morbidity and management of eye diseases and evaluated the gender differences in eye care in Oman.

## Materials and Methods

This was a retrospective review of the data collection instruments type of study. This data was generated in Oman through the following data sets: (1) two national surveys, (2) morbidity and management data, and (3) annual vision screening of school students. This review was conducted by the eye health care program at Muscat, Oman between June and December 2008.

Briefly, a population-based nationwide survey of Omanis (of all ages) was carried out in three months of 1996 - 97.[11] For this review, we included data on those aged 40 years and above. Bilateral blindness, cataract and aphakia/pseudophakia were calculated for men and women. Cataract was defined as lenticular opacity in the center that obstructs the view of the central fundus while examining with an ophthalmoscope through undilated pupil. Teams of doctors and nurses visited houses and tested vision using Snellen's distant vision chart. Those with vision less than 20/200 or showing defective confrontation test were reassessed in hospitals. An optometrist tested vision to determine the best corrected vision (BCVA) and ophthalmologists examined them to find out cause of visual disabilities. A person was defined as blind if BCVA was less than 20/400 or the field of vision was less than ten degrees.

**Figure 1:**
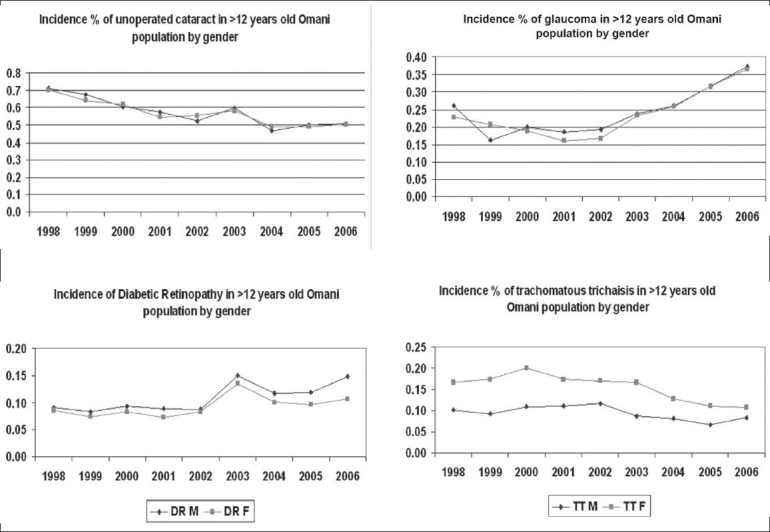
Rates of different blinding eye diseases in male and female adult Omani population (Reported by ophthalmologist at Ministry of Health Institutions from 1998 to 2007).

A similar nationwide survey was carried out in four months of 2005.[12] We used similar definitions of bilateral blindness, cataract and aphakia/ pseudophakia in two surveys. Although blindness due to uncorrected refractive error was available from the 2005 survey, we excluded the information on blindness related to uncorrected refractive error. Optometrists assessed vision while ophthalmologists examined eyes of participants. The flow of participants during the survey is given in Fig. 1. In, 1996 survey, all aged Omani population were surveyed while in the 2005 survey, 30 years and older Omani population were surveyed. To compare the data of two surveys, we calculated the prevalence of visual disabilities and eye diseases for all aged population in 2005. We assumed that rate of blindness in the less than 30-year-old population was similar in 1996 and 2005 in both sexes. In these surveys, inter-observer validation had kappa value of more than 0.8. Equipments were calibrated periodically. A pilot study was conducted. All investigators were trained in a standardization workshop.

Morbidity information that was as per the international classification of diseases (ICD 10) was collected and compiled from all regions.[13] The mid-year Omani population projections were taken as an index to calculate annual incidence rates.[10] The blinding eye conditions included cataract, trachomatous trichiasis (TT), glaucoma, and diabetic retinopathy. TT was defined as presence of eyelashes touching the eyeball and upper lid shows additional evidence of healed trachoma. Glaucoma was defined as combination of signs like (1) presence of glaucomatous changes of optic disc and surrounding retina, (2) presence of typical glaucomatous field changes tested on automated perimeter and/or (3) presence of ocular pressure of more than 22 mmHg (measured by applanation tonometry). Diabetic retinopathy was defined as presence of retinal changes typical of non-proliferative or proliferative grades in a person with diabetes. Management information included cataract surgeries, glaucoma surgeries, eyelid surgeries for TT and laser treatment for diabetic retinopathy. The difference of incidence of these blinding conditions in males and females along with their 95% confidence intervals were calculated.

The HIMS for eye diseases on monthly basis is managed by qualified health information officers. Nearly 95% of health services are catered to Omani citizens through MOH institutions at a cost of 2.5 dollars per year. The reviewed eye care data did not include institutes other than MOH hospitals.

The outcomes of vision screening of first, fourth, seventh, and tenth grade school students in 2005-06 were used to estimate the prevalence of different types of refractive error and the compliance of spectacle wearing, separately for boys and girls.

This data was compiled by qualified optometrists. Myopia was defined as having defective vision in either eye that improved after dynamic refraction and subjective correction with concave glasses of more than 0.5D power. Hypermetropia was defined as defective vision that improved with convex glasses of 0.5D or more following cycloplegic refraction. A student was considered as compliant if he/she was wearing spectacles on evaluation visit.

We calculated age and province-adjusted prevalence rates of male and females. We used parametric method of univariate analysis. We calculated Odd's ratio and its 95% confidence intervals by using EPI6 software (CDC, Atlanta).

To plot graphs, we used the number of male and female cases reported and surgeries done each year. Population projections for males and females were used as denominators for calculating incidence percentages of the blinding eye diseases. For indicators of their management, we calculated rates per million population.

## Results

The 1996 survey included 1,530 people of more than 40 years of age. The age-adjusted prevalence of bilateral blindness was 5.74% (95% CI 5.20 to 6.28) for men. It was 9.70% (95% CI 9.16 to 10.24) for women. Women were 1.21 times more likely to have unoperated cataract compared to men and 2.61 times more likely to have TT compared to men [Table 1]. The age-adjusted prevalence of blindness of men in the 2005 survey was 6.59%. (95% CI 5.09 to 8.14) compared to 9.93% (95% CI 8.34 to 11.52) among women. The 2005 survey revealed that gap of blindness prevalence between both sexes and other vision-related indices (cataract, and TT) was reduced. It reduced most prominently for TT (73% reduction in inequality), followed by cataract (51% reduction in inequality) and blindness (36% reduction in inequality).

**Table 1:** Prevalence of blindness and blinding eye diseases in males and females of > 40 years old Omani population (1996 and 2005)

	1996					2005				
		
	Male (n=728)		Female (n= 802)		odds ratio (95% CI) *P* value	Male (n=979)		Female (n= 1,360)		odds ratio (95% CI) *P* value	
	#	%	#	%		#	%	#	%	
Bilateral Blindness (Vision <20/400)	46	6.32	80	10.1	0.36 (0.24 -0.53) <0.001	75	7.66	107	7.87	0.97 (0.71 – 1.34) 0.91
Cataract	137	18.0	177	22.6	0.82 (0.63 – 1.03) 0.13	166	16.96	190	13.97	1.26 (1.00 – 1.59) 0.05
Trachomatous Trichiasis	41	5.28	123	15.7	0.33 (0.22 – 0.48) <0.001	32	3.27	66	4.85	0.66 (0.42 – 1.04) 0.07
Cataract surgical coverage* (%)	83		116		Difference 33%	78		61		Difference 17%

Cataract Surgery Coverage = Un-operated cataract/ (Aphakia + un-operated cataract) × 100 in patients with vision <20/400)

Analysis of blinding eye diseases revealed that incidence in men and women for most blinding eye conditions was not different [Table 2].

**Table 2:** Service delivery at Ministry of Health facilities in 1998 and 2005 by gender in Oman

Annual number of visits for specific eye diseases	Number of visits (per million 2005** population) in 1998*								

Male	Female	Difference	95% CI	Male	Female	Difference	95%	CI
Cataract	3,751 (0.14)	3,465 (0.14)	0.002	−0.01 to 0.01	3,155 (0.137)	3,055 (0.138)	0.002	−0.01 to 0.01
Glaucoma	1120 (0.07)	903 (0.08)	0.00	−0.01 to 0.01	2,014 (0.07)	1,981 (0.08)	0.003	−0.01 to 0.01
Diabetic Retinopathy (DR)	461 (0.03)	402 (0.03)	0.00	−0.01 to 0.00	753 (0.03)	599 (0.03)	−0.005	−0.01 to 0.00
Trachomatous Trichiasis (TT)	516 (0.02)	934 (0.03)	0.01	0.00 to 0.02	429 (0.02)	698 (0.03)	0.010	0.00 to 0.02
Annual number of surgeries (per million population)								
Cataract surgery	1,760 (0.20)	1,670 (0.19)	−0.01	−0.03 to 0.00	1330 (0.205)	1244 (0.194)	−0.011	−0.03 to 0.00
Glaucoma surgeries	188 (0.02)	200 (0.02)	0.00	0.00 to 0.01	116 (0.018)	122 (0.02)	0.001	0.00 to 0.01
Laser treatment for DR	248 (0.09)	187 (0.05)	−0.03	−0.04 to −0.02	559 (0.09)	349 (0.05)	−0.034	−0.04 to 0.02
Lid surgery for TT	163 (0.01)	323 (0.02)	0.01	0.01 to 0.01	54 (0.01)	129 (0.02)	0.010	0.01 to 0.01

* In 1998, the adult population comprised of 207,525 males and 311,740 females.

** In 2005, the adult population comprised of 534,732 males and 640,780 females. We used this data as the denominator for calculating annual incidence.

Indicator to review use of cataract surgical services, was ‘cataract surgical coverage’; the proportion of people who had surgery among those with operable cataract at a vision of less than 20/60. The data from both surveys revealed that for every man in need of cataract surgery there are 1.4 women in need of surgery.

**Figure 2:**
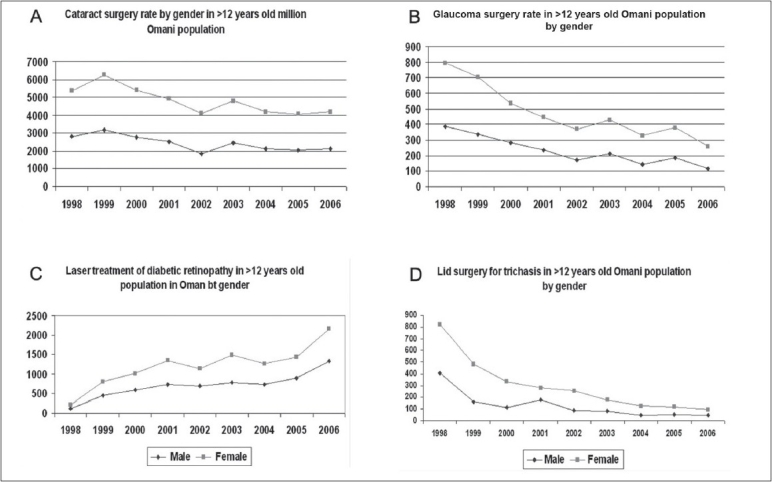
Eye surgeries per million male and female adult Omani population (1998 – 2007)

The cataract surgery rate (CSR) (number of surgeries per million among the ‘more than 12 years old’ Omani population per year) was the indicator used for reviewing cataract surgeries [Fig. 2 A]. Similar calculations were carried out for glaucoma surgeries [Fig. 2B], laser treatment of diabetic retinopathy [Fig. 2 C], and lid surgeries for TT [Fig. 2 D].

Myopia was rare (<0.5%) among first-grade (6 -7 years) students. The rate increased to 4.5% among seventh grade (12-13 years) students. The difference in prevalence between boys and girls of the tenth grade (16-17 years) was the most prominent. The compliance of spectacle wear was significantly higher in female students of tenth grade [Table 3].

**Table 3:** Refractive error and compliance of spectacle wear among school students in 2005-06 in Oman

	Boys	Girls	Difference	95% CI
1^st^ grade				
Examined	19,344	19,119		
Myopia	79	90		
Prevalence	0.41	0.47	0.6	−0.07 to 0.19
Hypermetropia	19	16		
Prevalence	0.10	0.08	0.02	−0.07 to 0.05
Compliance of spectacle wear	36/59	46/67		
	61.0	68.7	7.7	−0.9 to 24.4
4^th^ grade				
Examined	19,461	19,042		
Myopia	318	432		
Prevalence	1.63	2.27	0.64	0.36 to 0.91
Hypermetropia	48	45		
Prevalence	0.25	0.24	0.01	−0.11 to 0.09
Compliance of spectacle wear	147/220	180/276		
	66.4	68.1	1.8	−6.8 to 9.8
7^th^ grade				
Examined	22,590	21,080		
Myopia	895	1,093		
Prevalence	3.96	5.19	1.23	0.83 to 1.62
Hypermetropia	78	74		
Prevalence	0.35	0.35	0	−0.10 to 0.12
Compliance of spectacle wear	369/558	435/596		
	66.1	73.0	6.9	1.6 to 12.2
10^th^ grade				
Examined	23,108	19,638		
Myopia	1,472	1,967		
Prevalence	6.37	10.02	3.65	3.12 to 4.17
Hypermetropia	69	77		
Prevalence	0.30	0.39	0.09	−0.02 to 0.21
Compliance of spectacle wear	617/897	869/1,109		
	68.5	78.5	10.0	6.0 to 13.8

In 2005, Oman had 44 (30 male + 14 female) cataract surgeons in MOH. In addition, 32 ophthalmologists (23 male + 9 female) were working in eye units that do not have surgical facilities. Of 36 refractionists in MOH, nine were females and 27 were males.

## Discussion

This study focused on the gender equality issue in the eye care in Oman. The data of the year 1998 to 2000 represent the early period of the VISION 2020 initiative in Oman. Whereas the data of 2005 to 2007 could represent the effect of organized VISION 2020 strategies on reduction of blinding eye diseases in Oman. The positive impact in reduction of visual disabilities has been demonstrated in our earlier publication.[12] In the present study, we noted that females were at higher risk of blindness and blinding eye disease before implementing VISION 2020 strategies in Oman. The gender inequality reduced in the last few years and even became nonexistent for some eye conditions like blindness and TT. The eye care service utilization and the compliance of spectacle wear were also equal or even better among females.

In Oman, the proportion of males to females at birth is 51:49 but the life expectancy of women (74 years) is higher than that of men (72 years). Thus, the prevalence of age-related diseases is likely to be higher among women compared to men.[14] In addition, hormonal differences are likely to contribute to socio-cultural factors to increase female excess of TT.[15] The need for surgical services for cataract and TT for females would be 1.4 to 2.6 times more compared to that for men. Thus females seem to be victims of gender inequity in relation to the blinding and age related eye diseases. The study outcomes showed that the gender gap in eye care in Oman has reduced over time. The gender-sensitive annual data followed a set standard. Therefore, this review is less likely to be affected by misclassification or selection bias.

Kanthan *et al.* demonstrated that women had a significantly higher incidence of cataract than men.[16] The age-adjusted prevalence of cataract was also higher among women than among men in the United States (Odds Ratio = 1.37; 95% confidence interval, 1.26-1.50).[17] It seems that the industrialized countries with very high cataract surgery rates also have gender inequality causing more un-operated cataract in women compared to men. We did not find significant gender variation in the incidence of cataract reported in our hospitals. Less exposure to sunlight in Omani females due to less outdoor activities could explain fewer cataract cases in females.[18] The cataract surgical rate was 1.2-1.7 times higher for males than for females in India and China.[19] Addressing the gender inequality has been suggested to further reduce prevalence of un-operated cataract by 1.06% in Oman.[20]

The rate of diabetes mellitus (DM) among 30 to 65-year-old Omani males and females was 7.1% (95% CI6.2-8.1) and 5.1% (95% CI 4.4-6.0).[21] Even diabetic retinopathy (DR) was reported to be higher in Omani males than females.[22] Hence the higher prevalence of DR and laser treatment in males found in our study is logical.

The rate of eyelid surgery for TT is consistently higher among females compared to males in Oman. Proactive steps like case identification, health promotion and free of cost TT surgeries could have resulted in higher rates of TT surgeries in females.[23] The 2.6-fold excess prevalence of trichiasis found in women compared to men needs a gender-specific barrier study in Oman.

Glaucoma rates were consistently higher in males compared to females. It could be either due to higher prevalence of glaucoma in Omani males or more males with glaucoma seeking eye care.[24] Worldwide it is projected that by 2010, females will comprise 59% of all glaucoma cases.[25] Therefore, the program should focus more on the detection and care of glaucoma in females.

Eye condition based on screening of six to 16-year-old students focused on gender equality in the teenage population in Oman. Myopia was significantly higher in girls at four levels first, fourth, seventh and tenth grade.[26] Czecipta *et al.* also noted that gender influences the occurrence of myopia and hyperopia in 6-18-year-old children.[27] Aesthetic and visual needs could differ in boys and girls during their teenage years. Hence, female students had higher compliance rate for spectacle wear compared to male students of higher grades. This was in contrast to the findings of a study in Mexico where more boys were wearing spectacles compared to girls.[28] Less outdoor activities (sports) might be the reason for better compliance among Omani girls.

In many countries, HIMS are not gender-sensitive. As a result, program managers could not study the gender equality in eye care. Countries establishing HIMS should plan collecting data by gender.[5] It could be argued that changes in gender inequality noted in Oman in the last 12 years could be the reflection of overall socioeconomic development, improved health services and implementation of VISION 2020 in Oman.

The United Nation ranks member countries every year to review the status of gender equality. Oman was 119^th^ with ‘global gender gap index’ (GGGI) of 0.5903 and as per ‘health and survival’ sub-index (0.971), it was ranked 59^th^.[29] Thus, females seem to be underprivileged in Oman. Our study suggests that the gender inequality in eye diseases is not significant.

We compared the gender equality issue in eye care and GGGI of a few countries. We did not find any uniformity in the association of country's GGGI rankings and the gender equality for eye care. Germany with a score of 0.76 was ranked seventh. But, Rohrschneider found that more women were blind compared to men in Baden, Germany.[30] In Spain, ranked tenth with a score 0.744, Esteban *et al.* found that health-related quality of life was worse among women with visual impairment compared to men.[31] Iran with a score of 0.59, was ranked 118th. But the blindness rate in men (0.32%) was more than in women (0.25%) in Iran. This is contrast to previous examples of countries given higher rank and having females with higher rates of eye diseases.[32] Nepal (score of 0.557) was ranked 125th but rates of blindness were 1.2% in female and 1.1% in male.[33] Argentina (ranked 31^st^) had ‘Health and Survival sub-index’ of 0.98. In the same country, the cataract surgery rate in females (78%) was higher than in males (70%).[34] In Vietnam, (ranked 97th and sub-index of 0.97) blinding trachoma was more in females (8.9%) than in males (5.35%).[35] Thus, sub-indices of health matched with the gender equality in eye care but they could not explain GGGI. It seems that the factors affecting gender inequality at the national level and health services level are different and more complex compared to those working for eye care services. The indices of eye care will indirectly be affected by these factors. Therefore, by integrating gender inequality into its VISION 2020, avoidable blindness could be reduced in a country. But adopting a holistic approach targeting gender inequality beyond eye care and health would make it sustainable.

To determine gender equality in eye care services, standard indicators are yet not defined. In our study, we used gender-sensitive health information both at the community level and at eye care delivery sites. They provided trends of visual disabilities, blinding eye diseases, eye care service utilization, the compliance of spectacle wear, gender distribution of eye care providers and the client's choice of eye care provider's gender. Further studies are recommended using these indices. If they are found useful, they could be adopted to monitor the gender equality issue for eye care while monitoring the VISION 2020 initiative.

We observed a few limitations in our study. Population projections were based on two census data (1993 and 2003). If census methodologies differ, the prevalence would also differ. But we did direct standardization before comparing the data of the two surveys. The survey sample was calculated at the national level. Therefore, comparison of morbidity and management by gender at regional levels should be done with caution. In the last ten years, people of rural areas have migrated in Oman. Therefore, the prevalence based on the population in 2000 and 2007 would differ. If we assume that the migration was uniform in both genders, the bias will not affect the outcomes of our main objective of the study.

In our study, we found that a country having gender-sensitive health information enables the program staff to evaluate the gender inequality in eye care. The data of two surveys suggested that the gender gap in eye care had reduced in the last ten years. However, the hospital-based data seems to suggest that apart from glaucoma and TT surgeries, the difference in service utilization by gender was not statistically significant. Even though the prevalence of myopia was significantly higher in female students of higher classes, their compliance for spectacle wear was better.
